# Engineering Gut Symbionts: A Way to Promote Bee Growth?

**DOI:** 10.3390/insects15050369

**Published:** 2024-05-19

**Authors:** Pachara Sattayawat, Sahutchai Inwongwan, Nuttapol Noirungsee, Jilian Li, Jun Guo, Terd Disayathanoowat

**Affiliations:** 1 Department of Biology, Faculty of Science, Chiang Mai University, Chiang Mai 50200, Thailand; pachara.sattayawat@cmu.ac.th (P.S.); sahutchai.inwongwan@cmu.ac.th (S.I.); nuttapol.n@cmu.ac.th (N.N.); 2 Research Center of Deep Technology in Beekeeping and Bee Products for Sustainable Development Goals (SMART BEE SDGs), Chiang Mai University, Chiang Mai 50200, Thailand; 3 Institute of Apicultural Research, Chinese Academy of Agricultural Sciences, Beijing 100193, China; bumblebeeljl@hotmail.com; 4 Faculty of Life Science and Technology, Kunming University of Science and Technology, Kunming 650500, China; guojun0591@126.com

**Keywords:** honeybee, gut symbionts, genetic engineering, digestion, detoxification

## Abstract

**Simple Summary:**

Bees are important pollinators that play a role in balancing ecosystems; however, their survival rates have decreased due to many factors, including pathogens and exposure to pesticides. Bees have native mechanisms to help them tackle such challenges, and yet, these may not be enough. To this end, gut symbionts are beneficial, as they can help stimulate bees’ immune systems and detoxify ingested toxic chemicals. To enhance the efficiency of these mechanisms, genetic engineering is proposed in this work to further optimize the ability of bee gut symbionts, particularly in the dominant bacteria *Snodgrassella alvi* and *Gilliamella apicola*. Engineering strategies are discussed according to the gut symbiotic bacteria’s main roles in digestion, essential nutrient provision, and pesticide detoxification.

**Abstract:**

Bees play a crucial role as pollinators, contributing significantly to ecosystems. However, the honeybee population faces challenges such as global warming, pesticide use, and pathogenic microorganisms. Promoting bee growth using several approaches is therefore crucial for maintaining their roles. To this end, the bacterial microbiota is well-known for its native role in supporting bee growth in several respects. Maximizing the capabilities of these microorganisms holds the theoretical potential to promote the growth of bees. Recent advancements have made it feasible to achieve this enhancement through the application of genetic engineering. In this review, we present the roles of gut symbionts in promoting bee growth and collectively summarize the engineering approaches that would be needed for future applications. Particularly, as the engineering of bee gut symbionts has not been advanced, the dominant gut symbiotic bacteria *Snodgrassella alvi* and *Gilliamella apicola* are the main focus of the paper, along with other dominant species. Moreover, we propose engineering strategies that will allow for the improvement in bee growth with listed gene targets for modification to further encourage the use of engineered gut symbionts to promote bee growth.

## 1. Introduction

Bees are well-known for both their pollinator services, playing a crucial role in supporting economic crops and maintaining ecological balance, and their commercially high-value products. Currently, honeybee industries face many challenges, mostly related to the quality of bee products. For example, synthetic pesticides are toxic to bees and contaminate their products [1]. Overall, honeybee health is one of the key factors affecting the quality of bee products. It is known that the declining honeybee population is a result of various factors, including monoculture cropping [2], pesticide exposure [3], land fragmentation [4], mobile networks [5], and global warming [6]. Specifically, the use of pesticides has been shown to significantly reduce bee reproduction and, consequently, population growth. This includes both direct exposure of foraging bees and carryover effects [2]. Biotic factors also have a significant influence on honeybee populations, particularly in relation to the limited diversity within their gene pools [7] and their microbiome, which is associated with their health. It has been reported that a huge number of microorganisms impact bees’ health in positive and/or negative ways. Certainly, pathogenic microorganisms such as bacteria [8], fungi [9], and viruses [10] directly and negatively impact the health of honeybees, but non-pathogenic microorganisms are also important in altering their health. 

Symbiotic relationships between microbiota and insect hosts have been investigated and shown to be involved in several processes that benefit insect health, including tolerance to pathogens or parasites, and are also beneficial to their growth [11,12,13]. In temperate zones, winter is challenging, while in tropical areas, bees also contend with a dry season [14]. This results in the need to feed honeybees on supplementary food in these seasons. Gut symbionts are gut-dwelling microorganisms that maintain a commensal relationship with insects, by which the insects provide them with essential nutrients, and they fulfill the insect’s nutritional needs in return. Some vitamins, amino acids, monosaccharides, and fatty acids, for example, may not be consumed in forms that are ready to use by bees [15,16]; these are instead provided through gut bacterial metabolism. *Lactobacillus* is the most dominant genus of gut microbial community in bees, particularly *Lactobacillus* Firm-4 and *Lactobacillus* Firm-5 [17]. These strains have been shown to aid bee digestion [18] and adhere to the hindgut epithelium, which is an important anatomical part for nutrition utilization, like a protective film [19]. The film not only creates a conducive space but also serves as an anchor for another group of symbionts to establish a favorable station for habitation. Both of these functions contribute significantly to the honeybee’s ability to survive during challenging periods of the year. *S. alvi* is the second most dominant species of bee gut symbionts, along with *G. apicola* and *Bifidobacterium* [17]. Culturable strains of *S. alvi* have been isolated from honeybees (*Apis mellifera*) [20] and demonstrated to trigger bees’ response to pathogens [21]. *G. apicola* was also isolated in the same work. Interestingly, both of these bacteria are also symbionts to each other through metabolic cross-feeding, as shown from the analysis of their genomes [22]. *Bifidobacterium* accounts for a significant portion of the bee gut microbiome and also plays a role in aiding bee digestion [23]. Other roles of gut symbionts include detoxification of toxic substances ingested by insects [15,24] and stimulating the host’s immune responses against pathogens and parasites [25], which are essential for the overall health and survival of these organisms. Honeybee gut symbionts play a crucial role in preventing mortality from opportunistic pathogens such as *Serratia marcescens* [25] and *Hafnia alvei* [26], or parasites such as *Nosema ceranae* [27], as they act as host immunostimulants, through which they can induce the expression of antimicrobial peptides (AMPs) [28], thereby improving the survival rate of honeybees against these pathogens. In a reported case of the opportunistic pathogen *S. marcescens*, the gut microbiota played a role in eliminating pathogenic *S. marcescens* [25], although the exact mechanism has yet to be reported. 

It is clear that gut symbionts play a key role in promoting bee growth and survival. In this review, we explore engineering strategies that could potentially unlock the potential of gut symbionts even further, particularly helping to promote bee growth. Reported works and toolkits are also mentioned here to facilitate further investigations. Moreover, proposed engineering targets to promote bee growth via engineered gut symbionts, emphasizing their roles in aiding digestion through the overexpression of hydrolysis proteins and detoxification via the overexpression of detoxifying proteins, are listed for further research.

## 2. Engineering Gut Symbionts

Reengineering living organisms is logically driven by the desire to improve their native ability or to remove undesirable traits. Here, we collectively summarize all reported information and present it based on the purposes of each study. The first study on engineered gut symbionts, published in 2015, developed *L. kunkeei* as a potential bacterium for paratransgenesis [29], and since then, only a handful of works have been reported. *L. kunkeei* was transformed with *NucA* reporter gene controlled by an inducible promoter in order to develop genetic toolkits that can be applied to paratransgenesis. 

Later on, *S. alvi* was engineered to produce double-stranded RNA (dsRNA) as a stimulus for insect RNA interference (RNAi) defense responses to stimulate honeybee immunity and decrease infection from a wing-deforming virus and a parasite (*Varroa* mite) [21]. A recent work from the same group reported the use of engineered *S. alvi* to improve bee survival from a microsporidian parasite using a similar strategy. dsRNA was designed to target essential genes in *N. ceranae* [30]. The same invading parasite was also a target in another recent study, in which *S. alvi* was engineered to synthesize dsRNA corresponding to genes involved in the redox system of microsporidia [27]. 

Although the majority of studies on engineered *S. alvi* have involved increasing the survival rate of bees via stimulation of their immune responses towards pathogens or parasites, the use of gut symbionts as biosensors of the intestinal environment has recently been explored. In a recent work, *S. alvi* was engineered to detect the concentrations of IPTG and indicate these as fluorescent protein expression that can be detected in gut tissues or feces [31]. This is a prototypic biosensor that could be adapted to any environmental factor in a dose-dependent manner. 

Interestingly, *G. apicola*—the other dominant gut symbiont in bees—has also been engineered with reported synthetic biology toolkits [32], yet this bacterium has not been applied or used for any particular purposes. Other dominant symbionts, e.g., *Se. marcescens* N10A28 [31] and *B. apis* [32], have also been engineered and investigated thoroughly for compatible genetic parts and expression [32]. All reported works and conditions are summarized in Table 1.

The engineering of bee gut symbionts is advancing slowly due to several factors, one of which is the limited availability of genetic toolkits suitable for non-model microorganisms. Combining all genetic parts to form a new genetically functional system is the primary goal of synthetic biology studies. Firstly, gut symbionts’ regulatory elements may differ from those routinely used in model organisms and vice versa. Thus, the investigation and development of functional parts need to be implemented. A thorough study on the development of genetic toolkits for bee gut symbionts was published in 2018 [32]. This study screened several bio-bricks, facilitating further selection of each genetic part. A few years later, the same group published a thorough review article regarding the engineering of insect gut symbionts [33]. Indeed, it is a sound approach to begin by utilizing broad-host-range (BHR) plasmids when developing genetic toolkits for new bacterial strains, as there is a higher chance that the BHR machinery is universal and compatible with the machinery of the new target. However, the expression systems and selection of genetic parts are context-dependent. A highly functional system in one context, such as one strain/bacterium, may not always yield the same level of functionality in another context.

In a broader sense, it is indisputable that applications of genetically engineered organisms must be handled with caution and legally regulated. To this end, a significant challenge lies in thoroughly assessing the potential impacts of these organisms on bee populations and the broader environment. This rigorous testing process may necessitate time before practical implementation can be considered viable.

## 3. Proposed Engineering Strategies to Promote Bee Growth

The main roles of gut symbionts are either to promote bees’ digestion, to provide essential nutrients, or to detoxify ingested toxins. As mentioned previously, the engineering of bee gut symbionts has already been demonstrated to increase the survival rates of bees exposed to pathogens and parasites through stimulating bees’ immune responses rather than promoting bee growth through these basic roles. In addition to activating bee immunity, the engineered bacteria can be used as a stable delivery device for dsRNA. By selecting *Varroa* survival-related genes to design RNAi, dsRNA produced by bee intestinal engineered bacteria can induce *Varroa* survival-related gene silencing through *Varroa*’s feeding on bees, leading to *Varroa*’s death [34,35]. Here, we propose engineering strategies that potentially allow the promotion of bee growth via the enhancement of gut symbionts. The strategies are arranged based on their primary functions in digestion and nutrient provision and detoxification. Additional strategies are also outlined towards the end.

### 3.1. Digestion and Essential Nutrient Provision

Bees generally feed on flower pollen; however, the nutrients in this pollen may not be in a form that bees can directly utilize. Genome analysis of insect gut symbionts has unveiled their primary function as assisting in digestion by breaking down ingested nutrients and providing them in ready-to-use forms [12]. The increasing gene expression involved in such metabolism enhances the availability of nutrients and allows bees to obtain more from their diets, consequently promoting their growth. In this subsection, engineering strategies to aid digestion and nutrient provision are proposed.

**Figure 1:**
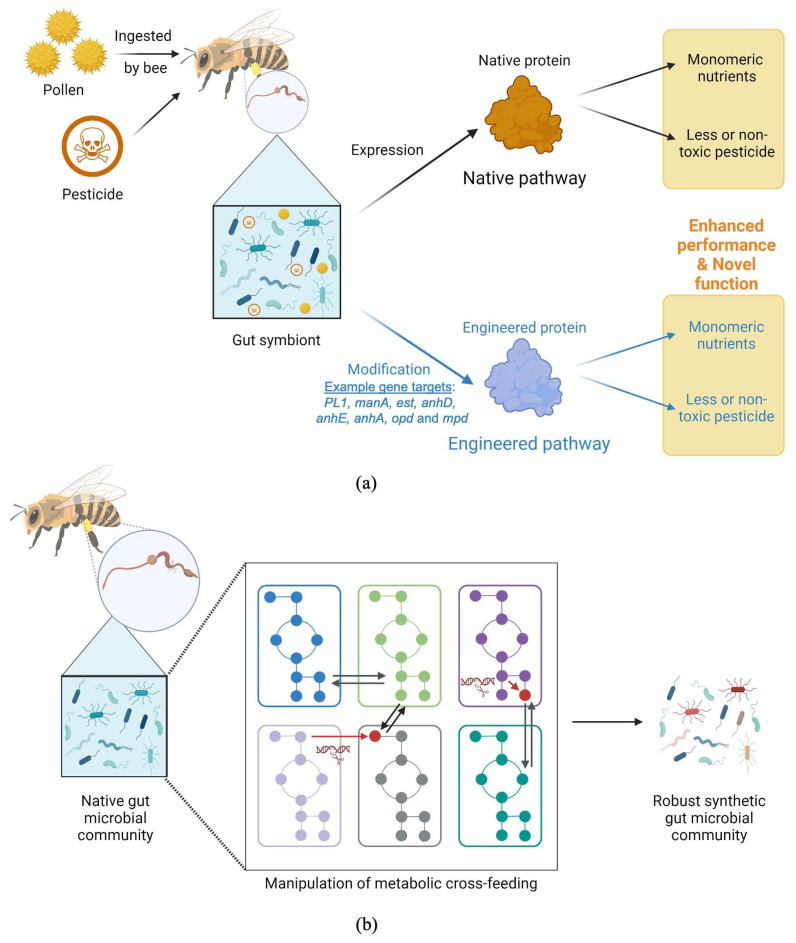
Proposed strategies to engineer bee gut symbionts for growth promotion through (**a**) expression of genes involved in digestion (i.e., *PL1* and *manA*) and pesticide detoxification (i.e., *est*, *anhD*, *anhE*, *anhA*, *opd* and *mpd*) and (**b**) manipulation of metabolic cross-feeding at community level.

Pectin is a major constituent in plant cell walls and bees cannot natively digest this substance. The ability to break down pectin relies on the presence of a pectin lyase gene (*PL1*) in gut microorganisms. *G. apicola* is known to contribute to pectin breakdown, but interestingly, not all isolated *G. apicola* strains harbor the *PL1* gene [15,36], leaving a gap in respect of strain improvements, i.e., introducing this gene into strains that do not naturally possess it (Table 2). This helps bees in two ways. First, degrading pectin facilitates pollen perforation, allowing bees to access nutrients. Second, pectin is considered a toxic carbohydrate for bees; degrading this compound enables bees to avoid its harmful effects and ensures their overall well-being. Short-chain fatty acids (SCFAs) produced through gut bacterial metabolism, especially that of *G. apicola* and *Lactobacillus*, have been shown to contribute to bee weight gain, with acetate and propionate being the most abundant SCFAs according to metabolomic analysis [37]. Given this information, improving SCFA synthetic pathways in these bacteria should allow more effective nutrient absorption and consequently enhance the growth of bees. *G. apicola* and *S. alvi* are two dominant species that have been predicted to exhibit metabolic cross-feeding. *G. apicola* utilizes carbohydrate fermentation, which yields fatty acids, along with glycolysis through the pentose phosphate pathway and Entner–Doudoroff pathway for energy production [22], and alternating these pathways for better performance could be a potential strategy. *S. alvi*, as a symbiont, utilizes the available oxygen in the gut, thus enabling the functional growth of *G. apicola*. Although the contribution may not be direct, improving the *S. alvi* oxygen utilization pathway may be an interesting choice. In addition, toxic sugars (i.e., mannose, xylose, arabinose, and rhamnose) are metabolized by gut symbionts, which results in improving dietary tolerance and promoting bee growth. The investigation of *G. apicola* as a toxic sugar degrader has revealed that all 42 isolated *G. apicola* strains from different bee species harbor mannose transporters, but not all contain a crucial metabolizing enzyme—mannose-6-phosphate isomerase (MPI)—encoded by the *manA* gene (Table 2). Ensuring the presence of this gene through genetic engineering would consequently promote mannose degradation. Degrading toxic sugars improves dietary tolerance in bees, and ultimately, their health. In the case of xylose, arabinose, and rhamnose, the presence of genes involved in their breakdown varies among different strains [15]. Engineering gut symbionts for their ability to break down these toxic sugars to usable carbon sources could allow relatively higher growth. Improving hydrolytic enzyme quantity and the quality of gut symbionts using genetic engineering could be a promising strategy towards improved digestion and nutrient uptake in bees (Figure 1a).

Moreover, metabolic models, particularly flux balance analysis (FBA) of the genome-scale metabolic model (GEM), relies on the stoichiometric properties of the network and optimizing solution of the objective function for the model to predict the flow rates of metabolites (metabolic flux) in a cell or between cells in a community. This metabolic phenotype depicts the integrative outcomes of all levels of regulation, including gene–protein reaction relationships, enabling the understanding of how the networks meet the demands of cells [38]. These computational techniques facilitate predicting and optimizing cellular metabolic fluxes, identifying bottlenecks, and exploration [39,40,41,42]. Increasingly, metabolic modeling is being employed to investigate the dynamics of microbial communities due to the advantages of in silico analysis over complex in vivo analyses of bacterial communities. The application of metabolic modeling approaches in engineering communities has been reviewed elsewhere [43], along with suggested strategies and available tools [44,45,46]. Utilizing GEMs, the metabolic functions of the human gut microbiome have been effectively investigated. Several successful examples of model-assisted metabolic engineering to increase metabolite production have been demonstrated in common hosts such as *Escherichia coli* and human gut symbionts [47,48,49]. For instance, the gut microbiome is implicated in metabolic disorders, including obesity and type 2 diabetes. Through analytical approaches and metabolic modeling, it has been observed that bacteria associated with these disorders exhibit increased consumption of glutamate, ammonia, arginine, and proline, as well as enhanced tartrate metabolism. These findings suggest that tartrate metabolism may be pivotal in microbiome-mediated metabolic alterations [50]. In addition, high-throughput genome-scale modeling suggests that microbial vitamin requirements influence the gut microbiome community structure. Using the GEMNAST framework, one study analyzed the metabolic needs of 816 gut strains, highlighting their vitamin dependencies and community interactions. Varied dependencies were observed among strains, with *Prevotella* strains complemented by other genera and Bacteroides strains showing prototrophic characteristics. Interaction modules between human and mouse gut species were identified. This research enhances the understanding of gut microbiome stability and health outcomes, and suggests potential applications of GEMNAST in nutritional studies [51]. Predictive models have indicated lactate and succinate cross-feeding. Cocultures exhibited enhanced carbohydrate utilization and consistent butyrate production. Xylan-derived supernatants reduced inflammation in HT-29 cells and protected Caco-2 cells from TcdB toxin, contrasting with inulin-derived supernatants. These findings emphasize the role of metabolic modeling and culture assays in understanding gut microbial interactions and potential health benefits [52]. GEMs offer a robust framework for investigating bee health by analyzing gut microbiome interactions and dynamics, paralleling established studies in human subjects. Despite the current lack of application to bee gut symbionts, implementing these predictive tools using a standardized workflow can effectively identify microbial engineering targets and enable theoretical studies of community dynamics. Integrating metabolic modeling approaches into synthetic biology holds significant potential for advancing the comprehensive and successful engineering of the bee gut microbiota.

While genome-scale metabolic network reconstruction has originally and commonly been based on individual genome data, recent advancements have extended these tools to the community level, enabling the prediction and identification of interactions between different species [41,44], including the extensively studied human gut microbiota [53,54]. Numerous tools have been developed for the automated construction of community-level metabolic models [49,55,56]. Community-scale metabolic models involve incorporating individual taxon-specific models within a larger dynamic framework, and as such, the assumption of a metabolic steady state used in regular flux balance analysis (FBA) may not apply. Dynamic flux balance analysis (dFBA) techniques, while computationally demanding and dependent on kinetic parameters, offer a continuous representation of changes from initial conditions to steady state. They have been effectively employed to model non-steady-state systems like microbial communities, demonstrating agreement with experimental data in identifying species interactions [57]. Additionally, approximation methods like SteadyCom and cooperative trade-off FBA (ctFBA) offer computationally feasible alternatives, with SteadyCom assuming equal growth rates for all taxa and ctFBA optimizing growth phases efficiently through trade-off considerations, both enabling refined solutions with reduced variability in the flux space [58,59].

The advances and current state-of-the-art in human gut community modeling have been extensively reviewed elsewhere [44,49,53,54] with several aspects investigated. In brief, studies tend to revolve around gut communities that are related to metabolic diseases. For example, community models have shown decreased synthesis of key metabolites such as acetate, butyrate, branch chain amino acids, lysine, tyrosine, and histidine in type 2 diabetes patients. Interestingly, individual models have predicted different scenarios where acetate and butyrate are produced in relatively higher amounts [50]. This emphasizes the fact that the gut microbial community functions as a cohesive system, with interactions between individual microorganisms contributing to larger-scale outcomes within the entire community. However, notable challenges involve discerning interactions, dynamic growth within the gut microbial population, obtaining accurate uncertain environmental data for constraints, and particularly the intricate task of validating metabolite-explicit models in complex scenarios. Nevertheless, the foundational principles of constructing and analyzing metabolic models for the human gut microbiome can certainly be extended to comparatively less complex communities, such as the bee gut microbiome. The bee gut contains a relatively simpler microbial community compared to that of humans. The metabolic interactions within the community have been proposed, and the genomes of identified bee microbiota are available [37,60,61]. Therefore, reconstructing the metabolic model of the bee gut microbiota and creating synthetic microbial communities or modifying the gut microbiota through metabolic engineering appear to hold higher potential and practicality than current studies in humans. However, the utilization of GEMs to model the microbiome necessitates validation, which can be accomplished in laboratory settings. The analysis of dynamic community composition can be conducted and monitored with metagenomic analysis. Additionally, metabolomic technology and individual species analysis are also highly feasible for the bee gut community species, given their relatively smaller number. Such information has been reported in several studies [61,62]. The potential applications of the bee gut community model align with those of the human gut microbiome model and, due to the simpler interactions and clear species roles, offer even more extensive opportunities. This paves the way for systems metabolic engineering or community engineering using synthetic biology approaches to enhance certain aspects of bee health (Figure 1b). Engineering the synthetic community of the gut microbiome based on the information obtained can then lead to an improved system, which has been demonstrated in the human gut. For example, in one study, a model-driven design for a microbial gut synthetic community was developed for high butyrate production [63], demonstrating that such synthetic communities are feasible and hold great potential for further development. 

### 3.2. Detoxification

This subsection centers primarily on the role of gut symbionts in pesticide detoxification, emphasizing pyrethroids, neonicotinoids, and organophosphates that pose risks to honeybees [64], and intentionally omits discussions on toxic compounds naturally present in plants. The potential role of bee symbionts in directly and indirectly reducing xenobiotic toxicity is highlighted by their response when exposed to such compounds. However, the literature on the biodegradation of xenobiotics by bee symbionts is limited. Consequently, the discussion here extends to taxa that are closely related to bee symbionts. This approach aims to leverage the known biodegradation mechanisms of related microorganisms and, through precise engineering, enhance or introduce these capabilities in bee symbionts. The listed detoxifying enzymes in this subsection are considered engineering targets to enhance xenobiotic detoxification efficiency. Ultimately, the overexpression or expression of detoxifying genes in engineered gut symbionts could enhance bee health through the mitigation of pesticide-induced toxicity. 

Pyrethroids have been used primarily as a pest control agent to manage mite infestations [65]. Exposure to insecticides in the pyrethroid family leads to changes in the relative abundance of core gut symbionts [66]. Intriguingly, in one study, *Serratia* emerged as one of the dominant members of the gut bacterial community, even though this genus is not typically counted as a core symbiont of *A. mellifera* [67]. The ability of *Serratia* to break down synthetic pyrethroids (beta-cypermethrin) has been established [68]. Nevertheless, *Serratia*’s role in bee pesticide detoxification remains undefined and warrants additional studies. The general chemical structure of pyrethroids is characterized by a cyclopropane ring, an alcohol moiety, and an ester linkage. The principal step in pyrethroid biodegradation is the hydrolysis of the carboxylester bond [69]. Carboxyesterase (EC 3.1.1.1) is the most studied enzyme involved in this step of microbial pyrethroid metabolism [70]. Prokaryotic genes responsible for pyrethroid degradation have been identified in both archaea, specifically in *Sulfolobus tokodaii* (*estSt7*) [71], and in bacteria. Pyrethroid-degrading genes have been found in bacteria belonging to the Alpha- and Gamma-proteobacteria classes, including *Klebsiella* (*estP*) [72]. Notably, strains of *Klebsiella*, isolated from the guts of *A. cerana*, have demonstrated the ability to inhibit *Paenibacillus larvae*, the causative agent of American foulbrood disease [73]. Consequently, engineering indigenous *Klebsiella* strains that suppress pathogens to also degrade pesticides holds potential for presenting dual benefits as bee probiotics (Table 2). Caution is needed when engineering pyrethroid-degrading symbionts due to mites’ high resistance to pyrethrin. Designing bacteria as an anti-mite-agent degrader is challenging due to the impracticality of eliminating the pest, though with potential benefits for bees in cases where higher doses of pyrethroid are needed. 

Neonicotinoids are a class of neuro-active insecticides. They are not directly used in apiculture, but they are extensively used in agriculture for pest control. Neonicotinoids can contaminate the pollen and nectar of treated plants, which are then collected by foraging bees [74]. Exposure to sublethal levels of thiamethoxam and imidacloprid results in a decreased abundance of *Bifidobacterium*, *Lactobacillus*, and *Lactobacillus* Firm-5. However, the population of *S. alvi* remains unaffected by these conditions. Conversely, thiamethoxam exposure leads to an uptick in the abundance of *G. apicola* [75]. In one study, after exposure to ecologically relevant doses of thiacloprid and acetamiprid, neither the growth of symbiotic isolates *in vitro* nor the gut microflora of honeybees was affected [76]. In another study, thiacloprid exposure influenced gut microbiome diversity in a dose-dependent manner. The abundance of *Lactobacillus* Firm-5 and *B. apis* saw a decline after exposure to thiacloprid. The impact on the microbiome was temporary, with core bacterial species recovering after 13 days of exposure, but the effect on honeybee survival persisted [77]. The resilience mechanisms of the symbiotic community and the role of symbionts in detoxification remain unexplored. Clothianidin exposure induces gut dysbiosis in *A. mellifera* [78]. Given the diverse molecular structures of these neonicotinoids, the metabolic pathways for neonicotinoid degradation are varied. The initial biotransformation reactions encompass a range of processes including deoxygenation, hydroxylation, oxidative cleavage, reduction, *N*-deacetylation, demethylation, and nitro reduction, implicating a variety of enzymes in these transformations. For instance, the nitrile hydratase (EC 4.2.1.84), encoded by the genes *anhD*, *anhE,* and *anhA* (Table 2), facilitates the transformation of the nitrile group of acetamiprid into an amide, a process undertaken by *Streptomyces canus* [79]. *Microvirga flocculans* is known to convert thiacloprid into a less toxic amide metabolite, a reaction mediated by nitrile hydratase enzymes encoded by *tnhA* and *thnB* genes [80]. Recent research has indicated that various species including *Edwardsiella, Serratia, Rahnella, Pantoea, Hafnia*, and *Enterobacter*, all isolated from the guts of honeybees, can degrade clothianidin in vitro [81]. However, it remains unknown whether these bacteria play a role in helping host bees counter the stress induced by clothianidin.

Organophosphate pesticides such as coumaphos and chlorpyrifos are widely used for apicultural and agricultural application [82]. In one study, exposure to coumaphos reduced the abundance of *Bifidobacterium* spp., *Lactobacillus* spp., and *Lactobacillus* Firm-5 [75]. However, chlorpyrifos did not significantly impact the dominant gut symbiont genera, including *Lactobacillus, Gilliamella, Snodgrassella, Commensalibacter, Bartonella*, and *Saccharibacter*, in either *A. mellifera* or *A. cerana* [83]. The most significant step in microbial degradation and detoxification is the hydrolysis of the ester bond. Initial hydrolysis of coumaphos under aerobic conditions yields diethylthiophosphate (DETP) and chloroferon [84]. The degradative pathway of chlorpyrifos also initiates the hydrolysis of the ester bond, resulting in DETP and 3,5,6-trichloro-2-pyridinol (TCP) [85]. Organophosphorus hydrolase (OPH, EC 3.1.8.1) is among the most extensively investigated organophosphorus degrading enzymes [86]. Two major families of hydrolases involved in bacterial organophosphorus metabolism are *opd*, encoding phosphotriesterase, and *mpd*, encoding the metallo-β-lactamase family [87] (Table 2). Microorganisms related to microflora associated with bees such as *Bacillus* [88], *Lactobacillus*, and *Serratia* have been shown to break down organophosphate pesticides [89,90,91,92]. 

Altogether, with the potential enzyme targets collectively listed here, the implementation of engineering is feasible, and the expression of the genes encoding detoxifying enzymes is the key (Figure 1a). Interestingly, a recent work showed that fungicides and insecticides affect honeybee cuticular microbiomes, suggesting that these chemicals can influence the microbiomes from other parts of the bee and not only the gut microbiome [93]. Thus, this can be considered an engineering target; however, knowledge in respect of the cuticle microbiome is still in its infancy. 

Examples of proposed engineering targets relating to primary roles of symbiotic bacteria are summarized in Table 2.

**Table 2:** Proposed engineering strategies of gut symbionts and targets to promote bee growth.

Main Role	Target (Gene or Metabolic Function)	Engineering Strategy	Expected Outcome	Reference
Digestion and nutrient provision	Pectin breakdown -*PL1* encoding pectin lyase	Overexpression	Improved pectin breakdown resulting in degraded toxic pectin to avoid intoxication and facilitate pollen perforation	[36]
	Mannose degradation -*manA* encoding mannose-6-phosphate isomerase	Overexpression	Improved mannose degradation resulting in degraded toxic mannose and improved bee dietary tolerance	[15]
Detoxification	Pyrethroid degradation -*est* encoding carboxyesterase	Expression	Improved pyrethroid degradation resulting in mitigation of pesticide-induced toxicity	[71,72]
	Neonicotinoid degradation -*anhD*, *anhE*, and *anhA* encoding nitrile hydratase	Expression	Improved neonicotinoid degradation resulting in mitigation of pesticide-induced toxicity	[79]
	Chlorpyrifos degradation -*opd* encoding phosphotriesterase -*mpd* encoding metallo-β-lactamase	Expression	Improved chlorpyrifos degradation resulting in mitigation of pesticide-induced toxicity	[87]

Please note that target bacteria can be any dominant symbiotic bacteria mentioned in the previous sections, but *S. alvi* and *G. apicola* could be interesting due to the available genetic tools and information.

## 4. Prospects in Gut Symbiont Engineering: Evaluating Strategies, Approaches, and Future Directions

In the previous sections, we provided information on reported genetic toolkits and proposed strategies to promote bee growth via engineering gut symbionts. Each strategy possesses advantages and disadvantages depending on the objective uses. Certainly, each strategy’s advantages include enhancing gut symbionts’ abilities according to the engineering purpose, which we anticipate will benefit bee health. The next question is what the disadvantages are. Generally, off-target effects may be one of the concerns as unintended genetic changes may occur during engineering. When these engineered symbionts are introduced to bee hosts, the outcomes may not be accurately foreseen. For instance, CRISPR-Cas9, although deemed compatible with engineering bee gut symbionts, carries the risk of off-target effects, potentially leading to unintended alterations in the genome [94]. The use of the plasmid system may be slightly more suitable in this case, as our proposed engineering strategies aim to express or overexpress particular genes. Plasmids, particularly broad-host-range plasmids, allow for straightforward customization of versatility, both in terms of the plasmid backbone and the selection of genes of interest for expression. Additionally, plasmids enable control over gene expression through the selection of plasmid genetic components, such as the origin of replication and promoters with available toolboxes [95]. Another concern, on a broader scale, pertains to the bee hosts and the environment. Ecological effects from the introduction of genetically modified microorganisms should not be overlooked. While microbial communities in bees have been observed to be structurally resilient to the introduction of new strains, there is still the possibility of disruption. Indeed, studies have demonstrated that introducing a new bacterial species can lead to community perturbation [96]. Moreover, the engineering strategies presented in this work should be validated to ascertain their efficacy in promoting bee growth. Key indicators for validation would include measurements of body weight and colony population size [97], serving as pivotal metrics for assessing the effectiveness of these strategies. Future developments that could ultimately and practically benefit bee health may involve the utilization of robustly engineered gut symbionts as probiotics. The use of probiotics derived from gut microbiomes has been recognized as an approach that can promote bee health and enhance their immune systems [98], which has been well-studied with commercially available products [99]. However, to date, although the concept of using engineered gut symbionts as probiotics, such as the successful engineering of *S. alvi* to enhance the immune system against pathogens [100], has been proposed, questions remain regarding the potential long-term effects on bee health or the environment. Therefore, conducting studies on extended exposure and comprehensive ecological assessments are imperative to fully understand the potential impacts and ensure the sustainability of these strategies in beekeeping practices.

## 5. Conclusions

This review has reported engineering toolkits for bee gut symbiotic bacteria, particularly in respect of the dominant species *S. alvi* and *G. apicola*. Proposed strategies to enhance bee growth by engineering their gut symbionts were discussed based on their main roles in aiding bee digestion and pesticide detoxification, paving the way for future research in this field. Overall, the overexpression of genes involved in nutrient breakdown, such as *PL1* (pectin lyase) and *manA* (mannose-6-phosphate isomerase), or pesticide degradation, such as *anhD, anhE,* and *anhA* (nitrile hydratase), is considered to be the low-hanging fruit strategy. Moreover, well-established engineering toolkits and strategies could lead to the development of robust symbiotic strains with desirable traits and, ultimately, the creation of bee probiotics for practical use. The expected potential of these strategies in respect of bee health and productivity can be divided into two aspects. To provide some examples, first, the avoidance of intoxication and the facilitation of pollen perforation from pectin breakdown enhance bee health and productivity by ensuring safer foraging conditions and efficient pollen utilization. Second, mitigation of pesticide-induced toxicity safeguards bee health and productivity by minimizing the harmful impact of pesticides on individual bees and colony dynamics, thereby supporting their long-term viability. Ultimately, the idea of using probiotics in bees has been discussed in a recent thorough review, including from the perspective of using engineered native gut symbionts [101], representing a promising avenue for future research. Extensive engineering strategies for enhancing overall bee growth have been proposed, although it is imperative to thoroughly investigate the long-term effects of using engineered bacteria. Additionally, as we work toward the development of a synthetic community housing engineered symbionts as bee probiotics, it is crucial to take into account legal regulations regarding the use of engineered bacteria as animal supplements. 

## Figures and Tables

**Table 1:** Reported studies on engineered bee gut symbionts.

Purpose	Symbiont	Outcome	Reference
Investigation of gut symbiosis and development of genetic toolkits for bee gut symbionts	*S. alvi*	Successful observation of bacterial colonization and developed bee microbiome toolkit (BTK) used for genetic engineering based on broad-host-range plasmid system (pRSF1010)	[32]
	*G. apicola*	Successful observation of bacterial colonization and developed bee microbiome toolkit (BTK) used for genetic engineering based on broad-host-range plasmid system (pRSF1010)	[32]
	*S. marcescens*	Successful observation of bacterial colonization and developed bee microbiome toolkit (BTK) used for genetic engineering based on broad-host-range plasmid system (pRSF1010)	[32]
	*B. apis*	Successful observation of bacterial colonization and developed bee microbiome toolkit (BTK) used for genetic engineering based on CRISPR system	[32]
Biosensor development for gut environment	*S. alvi*	Successful development of a biosensor system for fluorescence readouts through gut tissue and feces	[31]
Pathogen or parasite resistance	*L. kunkeei*	Successful transformation with no obvious adverse effects on honeybee survival	[28]
	*S. alvi*	Successful development of an engineered strain to induce RNAi mechanisms in honeybees for microsporidian parasite	[29]

## Data Availability

No datasets were generated or analyzed during the current study.

## References

[1] Wakgari M., Yigezu G. (2021). Honeybee Keeping Constraints and Future Prospects. Cogent Food Agric..

[2] St Clair A.L., St Clair A.L., Zhang G., Dolezal A.G., O’Neal M.E., Toth A.L., Toth A.L. (2020). Diversified Farming in a Monoculture Landscape: Effects on Honey Bee Health and Wild Bee Communities. Environ. Entomol..

[3] Stuligross C., Williams N.M. (2021). Past Insecticide Exposure Reduces Bee Reproduction and Population Growth Rate. Proc. Natl. Acad. Sci. USA.

[4] Brosi B.J., Daily G.C., Shih T.M., Oviedo F., Durán G. (2008). The Effects of Forest Fragmentation on Bee Communities in Tropical Countryside. J. Appl. Ecol..

[5] Halabi N.E., Achkar R., Haidar G.A. (2013). The Effect of Cell Phone Radiations on the Life Cycle of Honeybees. Proceedings of the Eurocon 2013.

[6] Settele J., Bishop J., Potts S.G. (2016). Climate Change Impacts on Pollination. Nat. Plants.

[7] Espregueira Themudo G., Rey-Iglesia A., Robles Tascón L., Bruun Jensen A., da Fonseca R.R., Campos P.F. (2020). Declining Genetic Diversity of European Honeybees along the Twentieth Century. Sci. Rep..

[8] Fünfhaus A., Ebeling J., Genersch E. (2018). Bacterial Pathogens of Bees. Curr. Opin. Insect Sci..

[9] Chen Y., Evans J.D. (2021). Honey Bee Fungal Diseases. Honey Bee Medicine for the Veterinary Practitioner.

[10] Yañez O., Piot N., Dalmon A., de Miranda J.R., Chantawannakul P., Panziera D., Amiri E., Smagghe G., Schroeder D., Chejanovsky N. (2020). Bee Viruses: Routes of Infection in Hymenoptera. Front. Microbiol..

[11] Raymann K., Moran N.A. (2018). The Role of the Gut Microbiome in Health and Disease of Adult Honey Bee Workers. Curr. Opin. Insect Sci..

[12] Jing T.Z., Qi F.H., Wang Z.Y. (2020). Most Dominant Roles of Insect Gut Bacteria: Digestion, Detoxification, or Essential Nutrient Provision?. Microbiome.

[13] Sinpoo C., In-On A., Noirungsee N., Attasopa K., Chantawannakul P., Chaimanee V., Phokasem P., Ling T.C., Purahong W., Disayathanoowat T. (2023). Microbial Community Profiling and Culturing Reveal Functional Groups of Bacteria Associated with Thai Commercial Stingless Worker Bees (*Tetragonula pagdeni*). PLoS ONE.

[14] Paray B.A., Kumari I., Hajam Y.A., Sharma B., Kumar R., Albeshr M.F., Farah M.A., Khan J.M. (2021). Honeybee Nutrition and Pollen Substitutes: A Review. Saudi J. Biol. Sci..

[15] Zheng H., Nishida A., Kwong W.K., Koch H., Engel P., Steele M.I., Moran N.A. (2016). Metabolism of Toxic Sugars by Strains of the Bee Gut Symbiont *Gilliamella Apicola*. MBio.

[16] Bonilla-Rosso G., Engel P. (2018). Functional Roles and Metabolic Niches in the Honey Bee Gut Microbiota. Curr. Opin. Microbiol..

[17] Kwong W.K., Moran N.A. (2016). Gut Microbial Communities of Social Bees. Nat. Rev. Microbiol..

[18] Su Q., Tang M., Hu J., Tang J., Zhang X., Li X., Niu Q., Zhou X., Luo S., Zhou X. (2022). Significant Compositional and Functional Variation Reveals the Patterns of Gut Microbiota Evolution among the Widespread Asian Honeybee Populations. Front. Microbiol..

[19] Vásquez A., Forsgren E., Fries I., Paxton R.J., Flaberg E., Szekely L., Olofsson T.C. (2012). Symbionts as Major Modulators of Insect Health: Lactic Acid Bacteria and Honeybees. PLoS ONE.

[20] Kwong W.K., Moran N.A. (2013). Cultivation and characterization of the gut symbionts of honey bees and bumble bees: Description of *Snodgrassella alvi* gen. nov., sp. nov., a member of the family *Neisseriaceae* of the *Betaproteobacteria*, and *Gilliamella apicola* gen. nov., sp. nov., a member of *Orbaceae* fam. nov., *Orbales* ord. nov., a sister taxon to the order ‘*Enterobacteriales*’ of the Gammaproteobacteria. Int. J. Syst. Evol. Microbiol..

[21] Horak R.D., Leonard S.P., Moran N.A. (2020). Symbionts Shape Host Innate Immunity in Honeybees: Symbionts Shape Honey Bee Immunity. Proc. R. Soc. B Biol. Sci..

[22] Kwong W.K., Engel P., Koch H., Moran N.A. (2014). Genomics and Host Specialization of Honey Bee and Bumble Bee Gut Symbionts. Proc. Natl. Acad. Sci. USA.

[23] Zheng H., Perreau J., Elijah Powell J., Han B., Zhang Z., Kwong W.K., Tringe S.G., Moran N.A. (2019). Division of Labor in Honey Bee Gut Microbiota for Plant Polysaccharide Digestion. Proc. Natl. Acad. Sci. USA.

[24] Wu Y., Zheng Y., Chen Y., Wang S., Chen Y., Hu F., Zheng H. (2020). Honey Bee (*Apis mellifera*) Gut Microbiota Promotes Host Endogenous Detoxification Capability via Regulation of P450 Gene Expression in the Digestive Tract. Microb. Biotechnol..

[25] Steele M.I., Motta E.V.S., Gattu T., Martinez D., Moran N.A. (2021). The Gut Microbiota Protects Bees from Invasion by a Bacterial Pathogen. Microbiol. Spectr..

[26] Lang H., Duan H., Wang J., Zhang W., Guo J., Zhang X., Hu X., Zheng H. (2022). Specific Strains of Honeybee Gut *Lactobacillus* Stimulate Host Immune System to Protect against Pathogenic *Hafnia alvei*. Microbiol. Spectr..

[27] Lang H., Wang H., Wang H., Zhong Z., Xie X., Zhang W., Guo J., Meng L., Hu X., Zhang X. (2023). Engineered Symbiotic Bacteria Interfering Nosema Redox System Inhibit Microsporidia Parasitism in Honeybees. Nat. Commun..

[28] Kwong W.K., Mancenido A.L., Moran N.A. (2017). Immune System Stimulation by the Native Gut Microbiota of Honey Bees. R. Soc. Open Sci..

[29] Rangberg A., Mathiesen G., Amdam G.V., Diep D.B. (2015). The Paratransgenic Potential of *Lactobacillus kunkeei* in the Honey Bee *Apis Mellifera*. Benef. Microbes.

[30] Yan X. (2023). Engineered Gut Symbiont Inhibits Microsporidian Parasite and Improves Honey Bee Survival. Proc. Natl. Acad. Sci. USA.

[31] Chhun A., Moriano-Gutierrez S., Zoppi F., Cabirol A., Engel P., Schaerli Y. (2024). An engineered bacterial symbiont allows noninvasive biosensing of the honey bee gut environment. PLoS Biol..

[32] Leonard S.P., Perutka J., Powell J.E., Geng P., Richhart D.D., Byrom M., Kar S., Davies B.W., Ellington A.D., Moran N.A. (2018). Genetic Engineering of Bee Gut Microbiome Bacteria with a Toolkit for Modular Assembly of Broad-Host-Range Plasmids. ACS Synth. Biol..

[33] Elston K.M., Leonard S.P., Geng P., Bialik S.B., Robinson E., Barrick J.E. (2022). Engineering Insects from the Endosymbiont Out. Trends Microbiol..

[34] Garbian Y., Maori E., Kalev H., Shafir S., Sela I. (2012). Bidirectional Transfer of RNAi between Honey Bee and *Varroa* Destructor: *Varroa* Gene Silencing Reduces *Varroa* Population. PLoS Pathog..

[35] Muntaabski I., Scannapieco A.C., Liendo M.C., Niz J.M., Russo R., Salvador R. (2022). Bacterially Expressed DsRNA Induces *Varroa* Destructor Gene Knockdown by Honey Bee-Mediated Oral Administration. J. Apic. Res..

[36] Engel P., Martinson V.G., Moran N.A. (2012). Functional Diversity within the Simple Gut Microbiota of the Honey Bee. Proc. Natl. Acad. Sci. USA.

[37] Zheng H., Powell J.E., Steele M.I., Dietrich C., Moran N.A. (2017). Honeybee Gut Microbiota Promotes Host Weight Gain via Bacterial Metabolism and Hormonal Signaling. Proc. Natl. Acad. Sci. USA.

[38] Kruger N.J., Ratcliffe R.G. (2015). Fluxes through Plant Metabolic Networks: Measurements, Predictions, Insights and Challenges. Biochem. J..

[39] Fang X., Lloyd C.J., Palsson B.O. (2020). Reconstructing Organisms in Silico: Genome-Scale Models and Their Emerging Applications. Nat. Rev. Microbiol..

[40] Gu C., Kim G.B., Kim W.J., Kim H.U., Lee S.Y. (2019). Current Status and Applications of Genome-Scale Metabolic Models. Genome Biol..

[41] Jansma J., El Aidy S. (2021). Understanding the Host-Microbe Interactions Using Metabolic Modeling. Microbiome.

[42] Zhang Z., Guo Q., Qian J., Ye C., Huang H. (2023). Construction and Application of the Genome-Scale Metabolic Model of *Streptomyces radiopugnans*. Front. Bioeng. Biotechnol..

[43] Li P., Roos S., Luo H., Ji B., Nielsen J. (2023). Metabolic Engineering of Human Gut Microbiome: Recent Developments and Future Perspectives. Metab. Eng..

[44] Esvap E., Ulgen K.O. (2021). Advances in Genome-Scale Metabolic Modeling toward Microbial Community Analysis of the Human Microbiome. ACS Synth. Biol..

[45] Ankrah N.Y.D., Bernstein D.B., Matthew B., Maureen C., Melinda E., Beatriz G.-J., Meiyappan L., Pacheco A.R., Snorre S., Medlock G.L. (2021). Enhancing Microbiome Research through Genome-Scale Metabolic Modeling. mSystems.

[46] Beura S., Kundu P., Das A.K., Ghosh A. (2022). Metagenome-Scale Community Metabolic Modelling for Understanding the Role of Gut Microbiota in Human Health. Comput. Biol. Med..

[47] Li Z., Gao C., Ye C., Guo L., Liu J., Chen X., Song W., Wu J., Liu L. (2023). Systems Engineering of *Escherichia coli* for High-Level Shikimate Production. Metab. Eng..

[48] Liu Y., Khusnutdinova A., Chen J., Crisante D., Batyrova K., Raj K., Feigis M., Shirzadi E., Wang X., Dorakhan R. (2022). Systems Engineering of *Escherichia coli* for N-Butane Production. Metab. Eng..

[49] Sen P., Orešič M. (2019). Metabolic Modeling of Human Gut Microbiota on a Genome Scale: An Overview. Metabolites.

[50] Proffitt C., Bidkhori G., Lee S., Tebani A., Mardinoglu A., Uhlen M., Moyes D.L., Shoaie S. (2022). Genome-Scale Metabolic Modelling of the Human Gut Microbiome Reveals Changes in the Glyoxylate and Dicarboxylate Metabolism in Metabolic Disorders. iScience.

[51] Molina Ortiz J.P., Read M.N., McClure D.D., Holmes A., Dehghani F., Shanahan E.R. (2022). High Throughput Genome Scale Modeling Predicts Microbial Vitamin Requirements Contribute to Gut Microbiome Community Structure. Gut Microbes.

[52] Belén H., Naschla G., Guillermo O., Marco V.-S., Pedro S., Martín G., Daniel G. (2022). Metabolic Modeling and Bidirectional Culturing of Two Gut Microbes Reveal Cross-Feeding Interactions and Protective Effects on Intestinal Cells. mSystems.

[53] Blasco T., Pérez-Burillo S., Balzerani F., Hinojosa-Nogueira D., Lerma-Aguilera A., Pastoriza S., Cendoya X., Rubio Á., Gosalbes M.J., Jiménez-Hernández N. (2021). An Extended Reconstruction of Human Gut Microbiota Metabolism of Dietary Compounds. Nat. Commun..

[54] Rios Garza D., Gonze D., Zafeiropoulos H., Liu B., Faust K. (2023). Metabolic Models of Human Gut Microbiota: Advances and Challenges. Cell Syst..

[55] Machado D., Andrejev S., Tramontano M., Patil K.R. (2018). Fast Automated Reconstruction of Genome-Scale Metabolic Models for Microbial Species and Communities. Nucleic Acids Res..

[56] Mendoza S.N., Olivier B.G., Molenaar D., Teusink B. (2019). A Systematic Assessment of Current Genome-Scale Metabolic Reconstruction Tools. Genome Biol..

[57] Mahadevan R., Edwards J.S., Doyle F.J. (2002). Dynamic Flux Balance Analysis of Diauxic Growth in *Escherichia coli*. Biophys. J..

[58] Chan S.H.J., Simons M.N., Maranas C.D. (2017). SteadyCom: Predicting Microbial Abundances While Ensuring Community Stability. PLoS Comput. Biol..

[59] Diener C., Gibbons S.M. (2023). More Is Different: Metabolic Modeling of Diverse Microbial Communities. mSystems.

[60] Kešnerová L., Mars R.A.T., Ellegaard K.M., Troilo M., Sauer U., Engel P. (2017). Disentangling Metabolic Functions of Bacteria in the Honey Bee Gut. PLoS Biol..

[61] Zhang Z., Mu X., Cao Q., Shi Y., Hu X., Zheng H. (2022). Honeybee Gut *Lactobacillus* Modulates Host Learning and Memory Behaviors via Regulating Tryptophan Metabolism. Nat. Commun..

[62] Liberti J., Kay T., Quinn A., Kesner L., Frank E.T., Cabirol A., Richardson T.O., Engel P., Keller L. (2022). The Gut Microbiota Affects the Social Network of Honeybees. Nat. Ecol. Evol..

[63] Clark R.L., Connors B.M., Stevenson D.M., Hromada S.E., Hamilton J.J., Amador-Noguez D., Venturelli O.S. (2021). Design of Synthetic Human Gut Microbiome Assembly and Butyrate Production. Nat. Commun..

[64] Sanchez-Bayo F., Goka K. (2014). Pesticide Residues and Bees—A Risk Assessment. PLoS ONE.

[65] FAO (2020). Good Beekeeping Practices: Practical Manual on How to Identify and Control the Main Diseases of the Honeybee (Apis mellifera).

[66] Dong Z.-X., Tang Q.-H., Li W.-L., Wang Z.-W., Li X.-J., Fu C.-M., Li D., Qian K., Tian W.-L., Guo J. (2022). Honeybee (*Apis mellifera*) Resistance to Deltamethrin Exposure by Modulating the Gut Microbiota and Improving Immunity. Environ. Pollut..

[67] Qi S., Al Naggar Y., Li J., Liu Z., Xue X., Wu L., El-Seedi H.R., Wang K. (2022). Acaricide Flumethrin-Induced Sublethal Risks in Honeybees Are Associated with Gut Symbiotic Bacterium *Gilliamella apicola* through Microbe-Host Metabolic Interactions. Chemosphere.

[68] Zhang C., Jia L., Wang S., Qu J., Li K., Xu L., Shi Y., Yan Y. (2010). Bio-degradation of Beta-Cypermethrin by Two *Serratia* spp. with Different Cell Surface Hydrophobicity. Bioresour. Technol..

[69] Maloney S.E., Maule A., Smith A.R. (1988). Microbial Transformation of the Pyrethroid Insecticides: Permethrin, Deltamethrin, Fastac, Fenvalerate, and Fluvalinate. Appl. Environ. Microbiol..

[70] Zhan H., Huang Y., Lin Z., Bhatt P., Chen S. (2020). New Insights into the Microbial Degradation and Catalytic Mechanism of Synthetic Pyrethroids. Environ. Res..

[71] Wei T., Feng S., Shen Y., He P., Ma G., Yu X., Zhang F., Mao D. (2013). Characterization of a Novel Thermophilic Pyrethroid-Hydrolyzing Carboxylesterase from *Sulfolobus tokodaii* into a New Family. J. Mol. Catal. B Enzym..

[72] Wu P.C., Liu Y.H., Wang Z.Y., Zhang X.Y., Li H., Liang W.Q., Luo N., Hu J.M., Lu J.Q., Luan T.G. (2006). Molecular Cloning, Purification, and Biochemical Characterization of a Novel Pyrethroid-Hydrolyzing Esterase from *Klebsiella* sp. Strain ZD112. J. Agric. Food Chem..

[73] Disayathanoowat T., Yoshiyama M., Kimura K., Chantawannakul P. (2012). Isolation and Characterization of Bacteria from the Midgut of the Asian Honey Bee (*Apis cerana* Indica). J. Apic. Res..

[74] Botías C., David A., Horwood J., Abdul-Sada A., Nicholls E., Hill E., Goulson D. (2015). Neonicotinoid Residues in Wildflowers, a Potential Route of Chronic Exposure for Bees. Environ. Sci. Technol..

[75] Rouzé R., Moné A., Delbac F., Belzunces L., Blot N. (2019). The Honeybee Gut Microbiota Is Altered after Chronic Exposure to Different Families of Insecticides and Infection by *Nosema ceranae*. Microbes Environ..

[76] Cuesta-Maté A., Renelies-Hamilton J., Kryger P., Jensen A.B., Sinotte V.M., Poulsen M. (2021). Resistance and Vulnerability of Honeybee (*Apis mellifera*) Gut Bacteria to Commonly Used Pesticides. Front. Microbiol..

[77] Liu Y.J., Qiao N.H., Diao Q.Y., Jing Z., Vukanti R., Dai P.L., Ge Y. (2020). Thiacloprid Exposure Perturbs the Gut Microbiota and Reduces the Survival Status in Honeybees. J. Hazard. Mater..

[78] El Khoury S., Gauthier J., Bouslama S., Cheaib B., Giovenazzo P., Derome N. (2021). Dietary Contamination with a Neonicotinoid (Clothianidin) Gradient Triggers Specific Dysbiosis Signatures of Microbiota Activity along the Honeybee (*Apis mellifera*) Digestive Tract. Microorganisms.

[79] Guo L., Fang W.W., Guo L.L., Yao C.F., Zhao Y.X., Ge F., Dai Y.J. (2019). Biodegradation of the Neonicotinoid Insecticide Acetamiprid by Actinomycetes *Streptomyces canus* CGMCC 13662 and Characterization of the Novel Nitrile Hydratase Involved. J. Agric. Food Chem..

[80] Zhao Y.-X., Jiang H.-Y., Chen X., Zhu Y.-X., Fan Z.-X., Dai Z.-L., Guo L., Liu Z.-H.L., Dai Y.-J. (2019). Neonicotinoid Thiacloprid Transformation by the N_2_-Fixing Bacterium *Microvirga flocculans* CGMCC 1.16731 and Toxicity of the Amide Metabolite. Int. Biodeterior. Biodegrad..

[81] El Khoury S., Giovenazzo P., Derome N. (2022). Endogenous Honeybee Gut Microbiota Metabolize the Pesticide Clothianidin. Microorganisms.

[82] Ravoet J., Reybroeck W., De Graaf D.C. (2015). Pesticides for Apicultural and/or Agricultural Application Found in Belgian Honey Bee Wax Combs. Bull. Environ. Contam. Toxicol..

[83] Yang Y., Ma S., Yan Z., Liu F., Diao Q., Dai P. (2019). Effects of Three Common Pesticides on Survival, Food Consumption and Midgut Bacterial Communities of Adult Workers *Apis cerana* and *Apis mellifera*. Environ. Pollut..

[84] Shelton D.R., Somich C.J. (1988). Isolation and Characterization of Coumaphos-Metabolizing Bacteria from Cattle Dip. Appl. Environ. Microbiol..

[85] Jabeen H., Iqbal S., Anwar S. (2015). Biodegradation of Chlorpyrifos and 3, 5, 6-Trichloro-2-Pyridinol by a Novel Rhizobial Strain *Mesorhizobium* sp. HN3. Water Environ. J..

[86] Singh B.K., Walker A. (2006). Microbial Degradation of Organophosphorus Compounds. FEMS Microbiol. Rev..

[87] Iyer R., Iken B., Damania A. (2013). A Comparison of Organophosphate Degradation Genes and Bioremediation Applications. Environ. Microbiol. Rep..

[88] Hayden C., Road E.A., Gilliam M. (2000). Identification and Roles of Non-Pathogenic Microflora Associated with Honey Bees. FEMS Microbiol. Lett..

[89] Cycoń M., Wójcik M., Piotrowska-Seget Z. (2009). Biodegradation of the Organophosphorus Insecticide Diazinon by *Serratia* sp. and *Pseudomonas* sp. and Their Use in Bioremediation of Contaminated Soil. Chemosphere.

[90] Islam S.M.A., Math R.K., Cho K.M., Lim W.J., Hong S.Y., Kim J.M., Yun M.G., Cho J.J., Yun H.D. (2010). Organophosphorus Hydrolase (OpdB) of *Lactobacillus brevis* WCP902 from Kimchi Is Able to Degrade Organophosphorus Pesticides. J. Agric. Food Chem..

[91] Armenova N., Tsigoriyna L., Arsov A., Petrov K., Petrova P. (2023). Microbial Detoxification of Residual Pesticides in Fermented Foods: Current Status and Prospects. Foods.

[92] Elzakey E.M., El-Sabbagh S.M., Eldeen E.E.S.N., Adss I.A.A., Nassar A.M.K. (2023). Bioremediation of Chlorpyrifos Residues Using Some Indigenous Species of Bacteria and Fungi in Wastewater. Environ. Monit. Assess..

[93] Schuhmann A., Sohl L., Thamm M., Scheiner R., Noll M. (2023). Fungicides and Insecticides Can Alter the Microbial Community on the Cuticle of Honey Bees. Front. Microbiol..

[94] Doudna J.A., Charpentier E. (2014). The New Frontier of Genome Engineering with CRISPR-Cas9. Science.

[95] Schuster L.A., Reisch C.R. (2021). A Plasmid Toolbox for Controlled Gene Expression across the Proteobacteria. Nucleic Acids Res..

[96] Shade A., Peter H., Allison S.D., Baho D.L., Berga M., Bürgmann H., Huber D.H., Langenheder S., Lennon J.T., Martiny J.B.H. (2012). Fundamentals of Microbial Community Resistance and Resilience. Front. Microbiol..

[97] Smart M., Pettis J., Rice N., Browning Z., Spivak M. (2016). Linking Measures of Colony and Individual Honey Bee Health to Survival among Apiaries Exposed to Varying Agricultural Land Use. PLoS ONE.

[98] Smriti, Rana A., Singh G., Gupta G. (2024). Prospects of Probiotics in Beekeeping: A Review for Sustainable Approach to Boost Honeybee Health. Arch. Microbiol..

[99] Damico M.E., Beasley B., Greenstein D., Raymann K. (2023). Testing the Effectiveness of a Commercially Sold Probiotic on Restoring the Gut Microbiota of Honey Bees: A Field Study. Probiotics Antimicrob. Proteins.

[100] Leonard S.P., Powell J.E., Perutka J., Geng P., Heckmann L.C., Horak R.D., Davies B.W., Ellington A.D., Barrick J.E., Moran N.A. (2020). Engineered Symbionts Activate Honey Bee Immunity and Limit Pathogens. Science.

[101] Motta E.V.S., Powell J.E., Leonard S.P., Moran N.A. (2022). Prospects for Probiotics in Social Bees. Philos. Trans. R. Soc. B Biol. Sci..

